# Synergistic Engineering of the Twin-Arginine Translocation (Tat) Pathway and Membrane Capacity Enhances Extracellular Production of Amylosucrase in *Bacillus licheniformis*

**DOI:** 10.3390/microorganisms13061179

**Published:** 2025-05-22

**Authors:** Caizhe Wang, Dandan Niu, Yongqing Zhou, Hui Liu, Nokuthula Peace Mchunu, Meng Zhang, Suren Singh, Zhengxiang Wang

**Affiliations:** 1 State Key Laboratory of Bio-Based Fiber Materials, Tianjin University of Science & Technology, Tianjin 300457, China; caizhewang@126.com (C.W.); yongqingzhou2022@163.com (Y.Z.); liuhui67788@163.com (H.L.); meng-zhang@tust.edu.cn (M.Z.); 2 Department of Biological Chemical Engineering, College of Chemical Engineering and Materials Science, Tianjin University of Science and Technology, Tianjin 300457, China; 3 National Research Foundation, P.O. Box 2600, Pretoria 0001, South Africa; np.mchunu@nrf.ac.za; 4 School of Life Science, University of KwaZulu Natal, Durban 4000, South Africa; 5 Department of Biotechnology and Food Science, Faculty of Applied Sciences, Durban University of Technology, P.O. Box 1334, Durban 4001, South Africa; singhs@dut.ac.za; 6 Tianjin Key Laboratory of Industrial Microbiology, Tianjin 300457, China

**Keywords:** amylosucrase, twin-arginine translocation (TAT) pathway, *Bacillus licheniformis*, extracellular enzyme secretion, membrane resource engineering, enzyme production

## Abstract

Amylosucrase (AS) is a highly versatile enzyme with significant potential for industrial applications, including functional food production and glycosylation of bioactive compounds. However, its large-scale production is hampered by low secretion efficiency in microbial hosts. This study focuses on engineering the twin-arginine translocation (TAT) pathway and optimizing membrane resource allocation in *Bacillus licheniformis* to enhance the extracellular production of *Neisseria polysaccharea* amylosucrase (NpAS). The investigation integrates three targeted strategies: optimizing the hydrophobic region adjacent to the TAT signal peptide, modifying TAT translocases via site-directed mutagenesis, and improving inter-pathway membrane resource redistribution by deleting non-essential Sec pathway components. Among the engineered strains, BLΔDF93S-2.0AS1 achieved an extracellular enzyme activity of 706.10 U/L, equating to a 2.01-fold improvement over the parental strain. These results emphasize the potential of combining multifaceted engineering strategies to optimize heterologous protein secretion systems.

## 1. Introduction

Amylosucrase (AS; EC 2.4.1.4) is a versatile enzyme capable of catalyzing the formation of consecutive α-(1,4)-O-glycosidic bonds to yield amylose and the isomerization to produce sucrose isomers such as turanose and trehalulose from its natural substrate sucrose [1,2,3]. Unlike many other glycosyltransferases, AS does not require costly sugar donors like adenosine diphosphate (ADP) or uridine diphosphate (UDP)-glucose [4], making it an attractive enzyme for industrial applications. Furthermore, recent studies have shown that AS can utilize sucrose as a donor molecule to release fructose and selectively transfer glucose to external biomolecules such as myricetin [4], isoquercitrin [5,6], and soybean isoflavone aglycones [7]. These unique catalytic properties highlight the enzyme’s potential for diverse applications, including functional food production, glycosylation of bioactive compounds, and carbohydrate synthesis.

However, the efficient production of AS by microbial strains remains a major challenge, limiting its large-scale industrial application. AS was initially discovered in the genus *Neisseria* [8], and subsequent studies have identified it in other bacterial genera, including *Deinococcus deserti* [9], *Calidithermus timidus* [10], *Deinococcus geothermalis* [11], *Truepera radiovictrix* [12], and *Bifidobacterium thermophilum* [13]. Despite its widespread occurrence, AS exhibits low activity levels in naturally isolated strains, typically around 50 U/L [12], which is far below the threshold required for industrial-scale production. This limitation has spurred efforts to explore heterologous expression systems as a means to enhance AS production. Among these, *Escherichia coli* has been the most extensively studied host for AS gene expression [14]. Early research primarily focused on identifying and characterizing AS-encoding genes, providing valuable insights into its genetic and biochemical properties, but achieving high expression levels was not a primary focus [15,16]. While optimization efforts in *E. coli* systems have led to progress [17,18], challenges such as host safety concerns, insufficient expression levels, and complex downstream processing remain significant barriers to large-scale applications [19].

Alternative hosts, such as *Bacillus subtilis* and yeast, have also been explored for AS expression. Notably, *B. subtilis* has been investigated for secretory expression of AS, but with limited success [20]. The underlying reasons for these challenges are not fully understood, pointing to the need for further research into optimizing secretion pathways in alternative microbial hosts. In our previous study, we successfully achieved the extracellular production of *Neisseria polysaccharea* amylosucrase (NpAS) in *Bacillus licheniformis* using the twin-arginine translocation (TAT) pathway. This resulted in an extracellular enzyme activity level of 350.45 U/L, accounting for 62.81% of the total enzyme activity [21]. While this represents a significant advancement over natural isolates, the expression level remains suboptimal for industrial applications and requires further improvement. The TAT pathway is regulated by three key determinants [22,23]: (1) the transfer efficiency of TAT translocases, (2) their membrane abundance, and (3) substrate-specific quality control mechanisms. Among these factors, membrane abundance of TAT translocases critically determines substrate translocation efficiency [24]. To improve pathway performance, genetic engineering strategies such as overexpression of TAT translocase components have been explored [25]. Additional approaches, including engineering N-terminal fusions of cargo proteins [26], and applying directed evolution to generate optimized TAT translocase variants [27], have also proven effective in increasing secretory protein yields via the TAT pathway. Nevertheless, the feasibility of these strategies for enhancing extracellular production of NpAS in *B. licheniformis* requires systematic validation.

This study aims to enhance the extracellular production of NpAS in *B. licheniformis* through targeted optimization of the TAT pathway. Specifically, we focus on three key strategies: (1) optimizing the retained mature peptide length following the TAT signal peptide to improve AS secretion, (2) modulating the expression levels of TAT translocase components, and (3) engineering the interplay between the TAT and Sec pathways to increase the spatial availability of TAT translocase in the cell membrane. By integrating these approaches, we achieved significant improvements in NpAS secretion, providing valuable insights into the engineering of extracellular protein production systems. Although the expression levels still fall short of industrial requirements, this work lays a solid foundation for future research and highlights promising directions for further optimization.

## 2. Materials and Methods

### 2.1. Strains, Plasmids, and Culture Conditions

The strains and plasmids used in this study are outlined in Table S1. *E. coli* JM109 and *B. subtilis* WB600 served as host strains for gene cloning and plasmid construction. *B. licheniformis* CBBD302B [21] was utilized as the parent strain for generating recombinant expression systems. For gene deletion experiments, the temperature-sensitive shuttle plasmid pUB-EX [28] was used. The plasmid pTAT1.0 [21], containing TAT-type *S_glmU_* signal peptide [29], served as the backbone for designing extracellular secretion systems. The *npas* gene (GenBank Accession No. Q9ZEU2), which encodes the amylosucrase from *N*. *polysaccharea*, was chemically synthesized by Sangon Biotech Co., Ltd. (Shanghai, China) and cloned into pTAT 1.0 or its derived plasmids for expression. Expression plasmids pWB-PulA and pHY-amyL, cloned gene encoding pullulanase (PulA) [30] or thermostable α-amylase (AmyL) [31], were used as controls to investigate the Sec-mediated secretion pathway.

All strains were cultivated at 37 °C in Luria–Bertani (LB) medium, which consists of 1% tryptone (Oxoid, Basingstoke, UK), 0.5% yeast extract (Oxoid, Basingstoke, UK), and 1% NaCl (Sinopharm Chemical Reagent Co., Ltd., Shanghai, China). Agar (1.5%) was added for solid media. To induce enzyme production, LB medium supplemented with 1% (*w*/*v*) lactose was employed. When necessary, the antibiotic kanamycin was added to a final concentration of 20 μg/mL.

### 2.2. Genetic Manipulation

#### 2.2.1. Standard Molecular Cloning Techniques

Standard molecular cloning techniques, including PCR amplification, plasmid extraction, restriction enzyme digestion, DNA ligation, and transformation into *E. coli*, were performed according to widely accepted protocols such as those described by Sambrook and Russell [32]. PCR primers utilized in the study are detailed in Table S1. All enzymes for molecular cloning were purchased from Takara Bio (Dalian, China) and used according to the manufacturer’s recommendations.

#### 2.2.2. Gene Deletion in *B. licheniformis*

Gene deletions were carried out using homologous recombination-mediated double-crossover methods [21]. First, the upstream and downstream flanking regions of the target gene were amplified by PCR from the genomic DNA of *B. licheniformis* CBBD302B. The two DNA fragments were subsequently fused via overlap PCR to create a deletion cassette. This cassette was inserted into pUB-EX [28] using *Bam*HI and *Xba*I restriction sites. The recombinant plasmid was then introduced into *B. licheniformis* via electroporation [31]. Transformants carrying the plasmid were selected for single-crossover integration by culturing at 40 °C, which prevents replication of the temperature-sensitive plasmid and facilitates chromosomal integration. PCR verification was performed at this stage using primers specific for the junctions between the plasmid and flanking genomic DNA. To induce double-crossover events for precise gene deletion, the strains were further incubated at 37 °C under non-selective conditions, allowing for loss of plasmid sequences. The resulting mutants were confirmed by diagnostic PCR using primers specific to regions flanking the deleted gene fragment. Details of primer sequences and amplification conditions are provided in Table S2.

#### 2.2.3. Prediction and Structure Analysis of NpAS Mutation Sites

Amylosucrase 1G5A (PDB: 1G5A) [33] was selected as the template for NpAS (GenBank Accession No. Q9ZEU2) homology modeling using software SWISS-MODEL Protein-modeling Server [34] (SWISS-MODEL Interactive Workspace (http://expasy.org/, accessed on 6 May 2025)). Structure comparison and analysis between NpAS and its mutants were performed by the program PyMOL [35] (Version 2.2.0, Schrödinger, LLC., New York, NY, USA).

#### 2.2.4. Site-Directed Mutagenesis of TAT Translocases

Site-directed mutagenesis was performed to introduce specific amino acid changes into TAT translocase-encoding genes. Overlap PCR was employed to generate mutant alleles [30]. For example, mutations such as TatAd L14Y were introduced by amplifying overlapping segments of the *tatAd* gene and fusing them into a full-length mutant gene. The mutant gene was integrated into the genome of the TAT translocase-defected strain (Table S1) using the homologous recombination methods described in Section 2.2.2. All other mutations were introduced following similar methodologies, with primers listed in Table S2. This approach allowed precise manipulation of TAT translocases to evaluate their impact on secretion efficiency.

### 2.3. Semi-Quantitative Enzyme Activity Analysis

Recombinant strains were assessed for extracellular enzyme activity using semi-quantitative plate-based assays. LB agar plates were supplemented with substrates corresponding to the target enzyme. For NpAS, plates contained 5% sucrose, and activity was visualized by iodine vapor staining after incubation [36]. For pullulanase (PulA), plates were supplemented with 1% pullulan, with hydrolysis zones revealed by ethanol treatment [37]. For α-amylase (AmyL), plates contained 1% starch to identify hydrolytic activity [28]. Plates were incubated at 37 °C for 12–36 h, depending on the enzyme expressed, to allow activity visualization.

### 2.4. Shaking Flask Fermentation

Shaking flask fermentations were conducted to assess enzyme production under controlled conditions [31]. Recombinant strains were inoculated into 50 mL of LB medium containing 1% lactose in 250 mL Erlenmeyer flasks at an initial *OD_600_* of approximately 0.1. Cultures were incubated at 37 °C and 200 rpm for up to 84 h. Bacterial growth was monitored by measuring the *OD_600_* of cell density at regular intervals, while extracellular enzyme activity was assessed from culture supernatants, as described in Section 2.5.

### 2.5. Enzyme Preparation and Activity Assays

#### 2.5.1. Sample Preparation

Culture supernatants were separated by centrifugation at 8000× *g* for 20 min at 4 °C to obtain the extracellular enzyme fraction. Cell pellets were washed twice with distilled water, resuspended in 50 mM Tris-HCl buffer (pH 7.0), and disrupted by ultrasonication (SCIENTZ-IID, Scientz, Ningbo, Zhejiang, China; output power 400 W, 20 × 3 s bursts, cooling on ice between cycles). The resulting lysates were clarified by centrifugation at 8000× *g* for 20 min at 4 °C. Both extracellular and intracellular samples were filtered through 0.45 μm filter membrane and stored at 4 °C until enzyme activity assays were performed. The molecular weight of NpAS was estimated based on SDS-PAGE [38], selecting 10% (*w*/*v*) running gel and 5% (*w*/*v*) stacking gel using the unstained protein molecular weight marker #26,610 (Thermo Fisher Scientific, Shanghai, China). Protein concentration was measured using the Micro Bradford method [39] using bovine serum albumin FV (Roche Diagnostics GmbH, Mannheim, Germany) as the standard.

#### 2.5.2. Activity Assays

NpAS Activity: NpAS activity was determined by incubating 0.1 mol/l sucrose in 50 mM Tris-HCl buffer (pH 7.0) with enzyme samples for 30 min at 35 °C [21]. The reaction was terminated by heating, and the amount of fructose released was quantified using the dinitrosalicylic acid (DNS) method [38]. One unit (U) of NpAS activity is defined as the amount of enzyme required to release 1 μmol of fructose per minute under assay conditions.

PulA and AmyL Activities: PulA activity was measured using pullulan as the substrate [30]. One unit (U) of PulA activity is defined as the amount of enzyme that produced one μmole of glucose reducing-sugars equivalents per minute under 60 °C and pH 4.5. AmyL activity assays utilized soluble starch [28]. One unit (U) of the AmyL activity was defined as the amount of enzyme needed to hydrolyze 1 mg soluble starch per minute at 70 °C and pH 6.0. These assays were used to assess the interaction of targeted secretion pathways with the Sec system.

## 3. Results and Discussion

### 3.1. Impact of Hydrophobic Region Modifications on NpAS Secretion in B. licheniformis

In the prior investigation, the secretion of NpAS in *B. licheniformis* was confirmed to be exclusively facilitated through the TAT pathway [21], utilizing the TAT-specific signal peptide S*_glmU_* [29], derived from glucosamine-1-phosphate N-acetyltransferase (GlmU). This system achieved a benchmark extracellular secretion efficiency of 62.81%, equating to an enzyme activity of 350.45 U/L [21]. Building upon this foundational study, further optimization strategies were evaluated. Previous research demonstrated that extending the hydrophobic region adjacent to the TAT signal peptide cleavage site could enhance protein secretion efficiency in certain cases. For instance, extending the hydrophobic region of proteins such as TorA has been reported to enhance their interaction with and recognition by TAT translocases, thereby improving translocation efficiency [26]. Inspired by these findings, we hypothesized that retaining downstream GlmU hydrophobic sequences might similarly enhance NpAS secretion in *B. licheniformis*.

To test this hypothesis, three constructs featuring N-terminal hydrophobic regions of varying lengths (45, 104, and 124 amino acids) derived from GlmU were engineered in-frame between S*_glmU_* and *npas* and designated as BL-45AS1, BL-104AS1, and BL-124AS1, respectively. Functional evaluation through sucrose-supplemented plates and shake flask fermentation demonstrated variable outcomes. Secretion efficiency exhibited a decline for all modified constructs compared to the unmodified reference strain [21]. Specifically, BL-45AS1 and BL-104AS1 showed extracellular enzyme activities of 60.35 U/L and 102.64 U/L, equating to secretion efficiencies of 37.61% and 40.45%, respectively. Notably, BL-124AS1 exhibited almost negligible extracellular activity, highlighting that over-elongated hydrophobic regions counterproductively impede secretion dynamics. While moderate hydrophobic region extensions yielded slight improvements over minimal designs, their overall secretion efficiency remained significantly lower than the 62.81% benchmark achieved without such modifications. These findings underscore that structural and functional incompatibilities within the TAT pathway may interfere with optimal secretion. Misrecognition or folding irregularities may play pivotal roles in limiting translocation.

Given these limitations, retaining hydrophobic region fragments as standalone fusion elements appears insufficient for enhancing NpAS secretion. Future investigations could explore avenues such as direct engineering of TAT translocases to improve substrate recognition and compatibility or integrating complementary pathways (e.g., Sec pathway) to mitigate bottlenecks, thereby unlocking the full potential of NpAS production for biotechnological applications.

### 3.2. Effect of TAT Pathway Modifications on NpAS Secretion Efficiency

#### 3.2.1. Influence of TAT Translocase Overexpression

**Figure 1:**
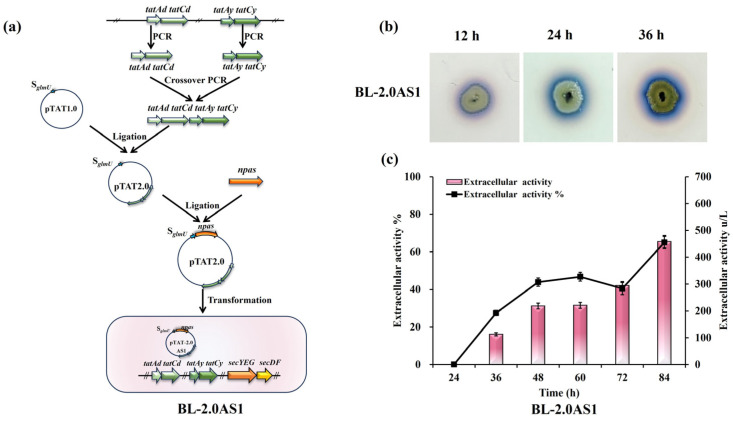
Effects of TAT translocase overexpression on NpAS secretion in *B. licheniformis*. (**a**) Engineering pipeline for the construction of BL-2.0AS1. (**b**) Temporal analysis of amylose biosynthesis: BL-2.0AS1 was cultivated on LB agar containing 5% sucrose, and amylose production was assessed at 12 h, 24 h, and 36 h using iodine vapor staining. (**c**) NpAS activity quantification: BL-2.0AS1 was grown in 250 mL Erlenmeyer flasks (50 mL working volume) at 37 °C and 200 rpm. Enzyme activity was measured in triplicate, with error bars representing standard deviation.

The biogenesis of the TAT transport system in *B. licheniformis* relies on two distinct translocase systems, TatAdCd and TatAyCy [25,40]. Overexpression of these components was posited as a potential avenue to improve NpAS secretion. To test this, supplemental copies of the *tatAdCd* and *tatAyCy* operons were introduced into strain *B. licheniformis* CBBD302B, generating strain BL-2.0AS1 (Figure 1a). Halo assays performed on sucrose-supplemented plates verified functional secretion of NpAS in BL-2.0AS1, with visible halo zones progressively enlarging over 12, 24, and 36 h of incubation (Figure 1b). Shake flask fermentations quantified a secretion efficiency of 65%, representing an extracellular enzyme activity of 458.05 U/L (Figure 1c). Compared to the benchmark strain’s 62.81% efficiency [21], this modest improvement highlights that merely increasing translocase availability is insufficient to fundamentally address secretion bottlenecks.

The limited enhancement suggests that intrinsic constraints, such as substrate recognition, folding fidelity, or the assembly rates of translocase complexes, may still impose limiting factors on secretion efficiency. More targeted approaches, including systematic mutagenesis of key functional translocase residues, could present opportunities for deeper functional optimization of TAT system behavior.

#### 3.2.2. Influence of TAT Translocase Mutations

**Figure 2:**
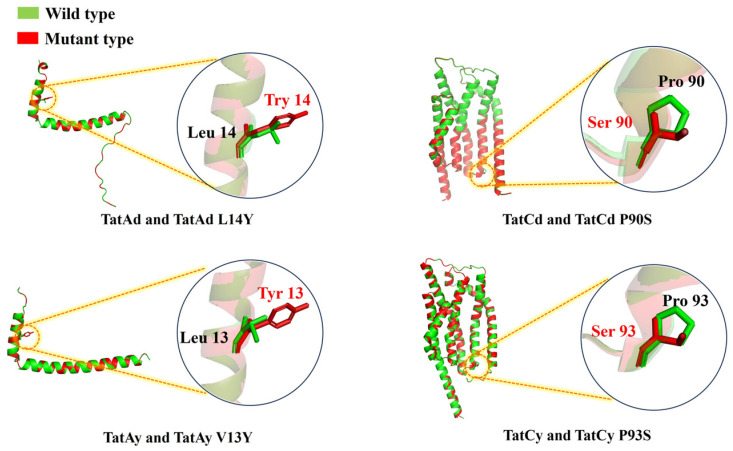
Structural schematic diagram comparison between the wild-type and the mutation sites of TAT translocases from *B. licheniformis.* Green: wild-type TAT translocase structure. Red: engineered TAT translocase variant with annotated amino acid substitution sites, as characterized in this study.

**Figure 3:**
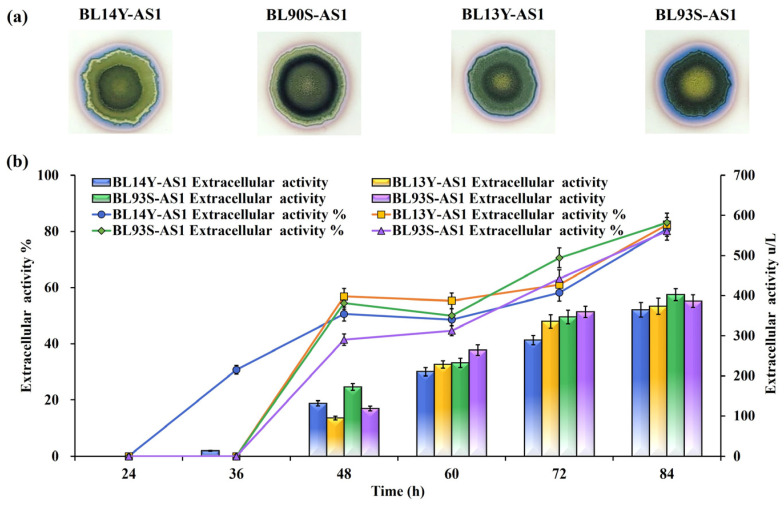
Effects of TAT translocase mutations on NpAS secretion in *B. licheniformis*. (**a**) Iodine vapor staining of strains on sucrose-supplemented agar plates at 12 h, 24 h, and 36 h. (**b**) NpAS volumetric activity in strains BL14Y-AS1, BL90S-AS1, BL13Y-AS1, and BL93S-AS1. Cultures were grown in 250 mL flasks (50 mL working volume) at 37 °C and 200 rpm for up to, 84 h. Error bars indicate standard deviation (n = 3).

To refine the functionality of TAT translocases, site-directed mutations were implemented based on structural insights into their roles in substrate recognition and translocation dynamics. Mutations introduced included TatAd L14Y, TatCd P90S, TatAy V13Y, and TatCy P93S (Figure 2), creating mutant strains BL14Y-AS1, BL90S-AS1, BL13Y-AS1, and BL93S-AS1, respectively. Halo assays revealed substantial secretion gains in all mutants, with BL13Y-AS1 exhibiting the most pronounced improvement (Figure 3a). Subsequent quantitative analysis showed enhanced secretion efficiencies: 82.42% for BL13Y-AS1, 80.11% for BL90S-AS1, 80.91% for BL14Y-AS1, and 83.11% for BL93S-AS1 (Figure 3b). These represent increases of 19.61%, 17.30%, 18.10%, and 20.30% over the original strain’s efficiency of 62.81% [21], significantly outperforming the enhancements achieved by translocase overexpression alone (65%).

Despite these promising gains in secretion efficiency, improvements in extracellular enzyme yield were relatively modest. The accelerated export facilitated by mutant translocases might have saturated the membrane’s capacity for protein translocation. This highlights the complex interplay between translocase function, substrate availability, and cell membrane resource allocation, indicating the need for systemic optimization across these interconnected factors. These results stress the potential of combining engineered translocases with broader cell membrane system interventions.

### 3.3. Enhancing TAT Pathway Efficiency Through Cell Membrane Resource Redistribution

Cell membranes are densely packed with proteins, essential for biological functions, despite their nanoscale size. With surface densities of about 30,000 proteins per square micrometer, each protein has only a few nanometers of space [41]. Bacterial cell membranes serve as dynamic platforms where multiple protein export pathways, including the TAT and Sec pathways, compete for finite membrane resources under macromolecular crowding constraints [42,43]. This nanoscale spatial limitation leaves each protein with only a few nanometers of available space, emphasizing the need for efficient resource allocation to maintain cellular function. To address these constraints, researchers have explored strategies such as promoting membrane surface folding and inhibiting FtsZ and MreB, which regulate cell division and peptidoglycan synthesis, to expand membrane area and enhance target protein production [44,45]. While membrane engineering is recognized as important, redistributing membrane resources to optimize pathway performance remains unexplored.

**Figure 4:**
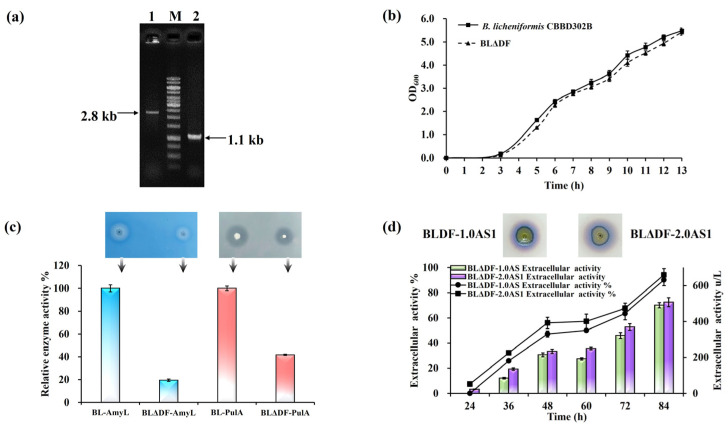
Sec pathway modification in *B. licheniformis*. (**a**) Colony PCR verification of *secDF* deletion: lane 1 (parent strain, 2.8 kb); lane 2 (BLΔDF, 1.1 kb). (**b**) Growth curves of CBBD302B and BLΔDF in LB medium (37 °C, 13 h). (**c**) Relative activity of AmyL and PulA in 250 mL flasks (50 mL working volume) at 37 °C and 200 rpm. Functional assays on LB plates: starch (1%) and pullulan (1%) hydrolysis by BLΔDF-AmyL and BLΔDF-PulA, respectively. The arrows indicate the corresponding strains. (**d**) NpAS volumetric activity in BLDF-1.0AS1 and BLDF-2.0AS1 (250 mL flasks, 50 mL, 37 °C, 200 rpm, 84 h). Iodine vapor staining at 36 h. Error bars: SD (n = 3).

To investigate whether redistribution of these resources could bolster TAT pathway performance, we selected the Sec-associated gene *secDF* for deletion. This gene is dispensable for cell viability [46], offering a viable target to reallocate membrane resources toward the TAT pathway. The mutant strain BLΔDF, generated through *secDF* deletion, was confirmed via PCR diagnostics and evaluated for physiological impacts (Figure 4a). Growth curves revealed no significant differences in viability compared to wild-type strains (Figure 4b). Functional assays using pullulanase and α-amylase as model Sec substrates showed declines in their secretion by 5.12-fold and 2.39-fold, respectively, indicating effective suppression of the Sec pathway (Figure 4c). Subsequent introduction of NpAS expression constructs into BLΔDF resulted in strains BLΔDF-1.0AS1 and BLΔDF-2.0AS1. Extracellular enzyme activities from these strains were 489.66 U/L and 506.86 U/L, respectively, corresponding to secretion efficiencies 25.37% and 26.52% higher than their respective baseline strains (Figure 4d). These findings demonstrate the viability of mitigating inter-pathway competition to enhance the efficiency of TAT-dependent secretion, aligning with broader goals of optimizing resource allocation across membrane-bound systems.

Attempts to delete other Sec components (SecY, SecE, and SecG) were unsuccessful, highlighting their necessity for bacterial survival. Future studies could explore conditional knockdowns or novel genetic strategies to more precisely modulate Sec-TAT interactions. These approaches could build on the insights gained here, offering refined platforms for increasing the efficacy of extracellular protein secretion.

### 3.4. Enhanced Secretion of NpAS Through Combined Regulation Strategies

**Figure 5:**
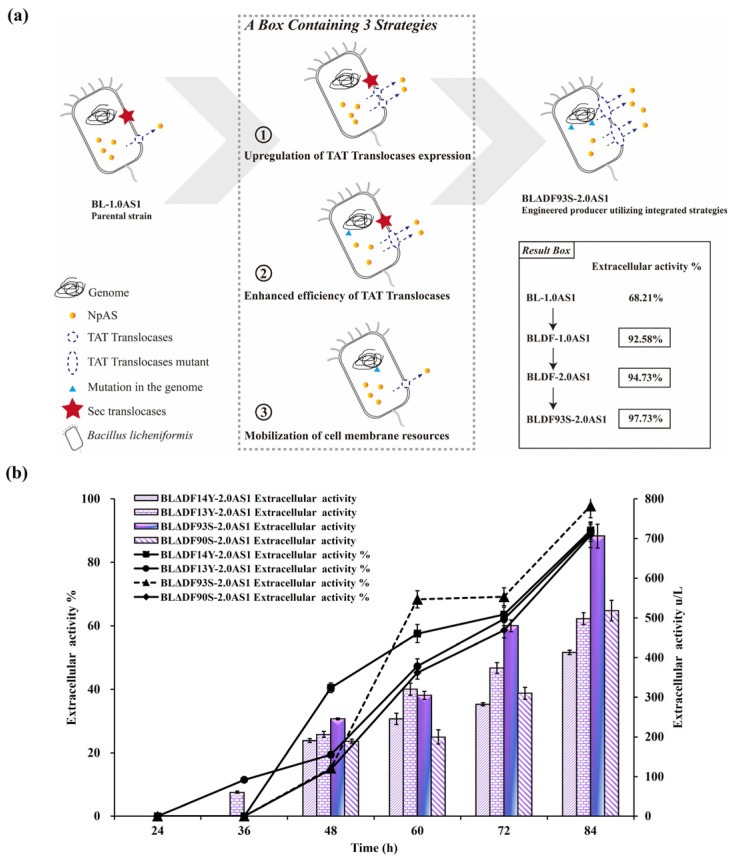
Effects of combined regulation strategies coordinated on the secretion of NpAS in *B. licheniformis*. (**a**) The engineering approach for constructing an NpAS production strain. The result box highlights the increased secretory capability of NpAS, achieved via synergistic engineering strategies. (**b**) NpAS volumetric activities in strain BLΔDF14Y-2.0AS1, BLΔDF13Y-2.0AS1, BLΔDF90S-2.0AS1, and BLΔDF93S-2.0AS1 under shake flask fermentation testes. Error bars: SD (n = 3).

**Figure 6:**
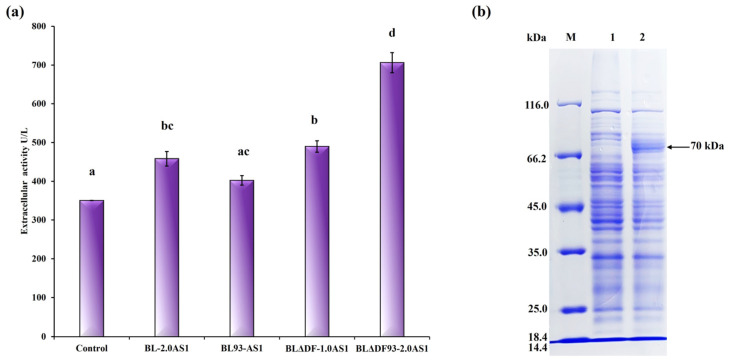
Summary of the influence of different engineering strategies on the secretion of NpAS in *B. licheniformis*. (**a**) NpAS volumetric activity in BL-2.0AS1 (upregulation of TAT translocases expression), BL93-AS1 (enhanced efficiency of TAT translocases), BLΔDF-1.0AS1 (mobilization of cell membrane resources), BLΔDF93S-2.0AS1 (the combination of three strategies). The letters above the bars indicate statistical differences found by a Mann–Whitney U test (*p* < 0.001). (**b**) SDS-PAGE analysis of culture supernatant by strain BLΔDF93S-2.0AS1. M: protein molecular weight marker (*E. coli* β–galactosidase 116 kDa, bovine serum albumin 66.2 kDa, chicken egg ovalbumin 45 kDa, porcine lactate dehydrogenase 35 kDa, *E. coli* REase Bsp98I 25 kDa, bovine milk β–lactoglobulin 18.4 kDa, chicken egg lysozyme 14.4 kDa); 1: extracellular supernatant of parental strain; 2: extracellular supernatant of BLΔDF93S-2.0AS1.

To achieve the highest possible enhancement of NpAS secretion, a combined regulatory approach was employed. By integrating strategies including *secDF* deletion, TAT translocase overexpression, and site-specific mutations, we implemented a comprehensive optimization pathway (Figure 5a). Among the constructed strains, BLΔDF93S-2.0AS1 emerged as the most efficient, achieving an extracellular enzyme activity of 706.10 U/L (total activity 722.45 U/L) with a secretion efficiency of 97.74% (Figure 5b). This corresponds to a 2.01-fold improvement compared to that of BL-1.0AS1, which showed extracellular activity of 350.45 U/L (total activity 557.92 U/L) with a secretion efficiency of 62.81% [21]. Notably, the result reveals that the engineered recombinant strain not only achieved superior secretion efficiency but also maintained enhanced total protein expression capacity. This significant advance confirms the synergistic potential of combining multiple genetic modifications to mitigate TAT pathway limitations. Significantly, this combined approach outperformed single strategies. While *secDF* deletion alone achieved 506.86 U/L (BLΔDF-2.0AS1), the integration of functional mutations and translocase overexpression resulted in superior performance in BLΔDF93S-2.0AS1 (Figure 6a) and successfully transported the active NpAS into the culture media (Figure 6b). This demonstrates the necessity of addressing both quantitative membrane dynamics and qualitative translocase functionality to overcome bottlenecks.

These results highlight the imperative for comprehensive strategies to optimize protein secretion systems. By simultaneously addressing cell membrane resource allocation, translocase efficiency, and substrate recognition, the integrated approaches explored herein provide a robust framework for achieving industrially relevant levels of secretion. These findings pave the way for the scalable use of TAT-dependent secretion systems in biotechnological applications.

## 4. Conclusions

This study achieved significant enhancement in NpAS secretion in *B. licheniformis* by optimizing the TAT pathway through translocase overexpression, targeted mutations, and *secDF* deletion to redistribute membrane resources. The highest extracellular enzyme activity, 706.1 U/L, was achieved in strain BLΔDF93S-2.0AS1, representing a 2.01-fold improvement over the baseline strain. These results highlight the potential of integrated genetic and resource-engineering strategies for scalable heterologous protein production, paving the way for further refinement of bacterial secretion systems for industrial applications.

## Data Availability

The original contributions presented in this study are included in the article/Supplementary Materials. Further inquiries can be directed to the corresponding authors.

## Supplementary Materials

The following supporting information can be downloaded at: https://www.mdpi.com/article/10.3390/microorganisms13061179/s1, Table S1: Strains and plasmids used in this study; Table S2: Nucleotide sequence of primers.

## Abbreviations

The following abbreviations are used in this manuscript:

AS	Amylosucrase
ADP	Adenosine diphosphate
UDP	Uridine diphosphate
NpAS	*Neisseria polysaccharea* amylosucrase
LB	Luria–Bertani medium
PCR	Polymerase chain reaction
DNS	Dinitrosalicylic acid
