# Supplementary material for: Synergistic Engineering of the Twin-Arginine Translocation (Tat) Pathway and Membrane Capacity Enhances Extracellular Production of Amylosucrase in Bacillus licheniformis
Source: Microorganisms. 2025 May 22;13(6):1179. doi: 10.3390/microorganisms13061179 (PMC12195555; doi:10.3390/microorganisms13061179)
Supplement: Supplementary file 1 [file microorganisms-13-01179-s001.zip › microorganisms-3552629-supplementary.pdf]

Table S1. Strains and plasmids used in this study

Strain/Plasmid	Characteristics	Source
**Strain**		
E. coli JM109	*endA1*, *recA1*, *gyrA96*, *thi*, *hsdR17*, *rel*A1, *sup*E44, λ⁻, Δ(*lac-proAB*), [F′, *traD36*, *proAB*, *laqI*^{q}ZΔM15]	Lab stock
B. subtilis WB600	*B. subtilis* 168, Δ*nprE*, Δ*aprA*, Δ*epr*, Δ*bpf*, Δ*mpr*, Δ*nprB*	Lab stock
B. licheniformis CBBD302B	Host cell for gene expression	[21]
BL-45AS1	*B. licheniformis* CBBD302B harboring pTAT1.0-45AS1	This study
BL-104AS1	*B. licheniformis* CBBD302B harboring pTAT1.0-104AS1	This study
BL-124AS1	*B. licheniformis* CBBD302B harboring pTAT1.0-124AS1	This study
BL-1.0AS1	*B. licheniformis* CBBD302B, harboring pTAT1.0-AS1	[21]
BL-2.0AS1	*B. licheniformis* CBBD302B, harboring pTAT2.0-AS1	This study
BLΔACd	Derived from *B. licheniformis* CBBD302B, ΔtatAd-tatCd	This study
BLΔACy	Derived from *B. licheniformis* CBBD302B, ΔtatAy-tatCy	This study
BL14Y	*B. licheniformis* CBBD302B, mutation of L14Y in TatAd	This study
BL90S	*B. licheniformis* CBBD302B, mutation of P90S in TatCd	This study
BL13Y	*B. licheniformis* CBBD302B, mutation of V13Y in TatAy	This study
BL93S	*B. licheniformis* CBBD302B, mutation of P93S in TatCy	This study
BLΔDF	*B. licheniformis* CBBD302B, Δ*secDF*	This study
BLΔDF-PulA	*B. licheniformis* CBBD302B with the deletion of the *secDF* gene and harboring pWB-PulA	This study
BL-PulA	*B. licheniformis* CBBD302B harboring pWB-PulA	This study

BLΔDF-AmyL	*B. licheniformis* CBBD302B with the deletion of the *secDF* gene and harboring pHY-AmyL	This study
BL-AmyL	*B. licheniformis* CBBD302B harboring pHY-AmyL	This study
BLΔDF-1.0AS1	BLΔDF, harboring pTAT1.0-AS1	This study
BLΔDF-2.0AS1	BLΔDF, harboring pTAT2.0-AS1	This study
BLΔDF14Y	*B. licheniformis* CBBD302B, Δ*secDF*, mutation of L14Y in TatAd	This study
BLΔDF90S	*B. licheniformis* CBBD302B, Δ*secDF*, mutation of P90S in TatCd	This study
BLΔDF13Y	*B. licheniformis* CBBD302B, Δ*secDF*, mutation of V13Y in TatAy	This study
BLΔDF93S	*B. licheniformis* CBBD302B, Δ*secDF*, mutation of P93S in TatCy	This study
BLΔDF14Y-2.0AS1	BLΔDF14Y, harboring p14Y-2.0AS1	This study
BLΔDF90S-2.0AS1	BLΔDF90S, harboring p90S-2.0AS1	This study
BLΔDF13Y-2.0AS1	BLΔDF13Y, harboring p13Y-2.0AS1	This study
BLΔDF93S-2.0AS1	BLΔDF93S, harboring p93S-2.0AS1	This study
**Plasmid**		
pUB-EX	Km^R, thermosensitive plasmid, harboring the expression cassette of pHY-WZX	[28]
pWB-PulA	Km^R, carrying the pullulanase-encoded gene from *B. naganoensis* ATCC 53,909	[30]
pHY-AmyL	Km^R, carrying the *α*-amylase encoded gene from *B. licheniformis*	[28]
pTAT1.0-AS1	Harboring S_{glmU} from *B. licheniformis*, *npas* gene, Km^R	[21]
pTAT1.0	Harboring S_{glmU} from *B. licheniformis*, Km^R	[21]
pTAT1.0-45	Harboring S_{glmU} from *B. licheniformis*, the first 45 amino acid gene of GlmU, Km^R	This study

pTAT1.0-104	Harboring S_{glmU} from *B. licheniformis*, the first 104 amino acid gene of GlmU, Km^R	This study
pTAT1.0-124	Harboring S_{glmU} from *B. licheniformis*, the first 124 amino acid gene of GlmU, Km^R	This study
pTAT1.0-45AS1	Harboring S_{glmU} from *B. licheniformis*, the first 45 amino acid gene of GlmU, *npas* gene, Km^R	This study
pTAT1.0-104AS1	Harboring S_{glmU} from *B. licheniformis*, the first 104 amino acid gene of GlmU, *npas* gene, Km^R	This study
pTAT1.0-124AS1	Harboring S_{glmU} from *B. licheniformis*, the first 124 amino acid gene of GlmU, *npas* gene, Km^R	This study
pTAT2.0	Harboring *tatAdCd* and *tatAyCy* gene in pTAT1.0, Km^R	This study
pTAT2.0-AS1	Harboring *npas* gene in pTAT2.0, Km^R	This study
pUB-*secDF*	Harboring *B. licheniformis secDF* deletion cassette, Km^R	This study
p14Y-2.0	Harboring *tatAdCd*-14Y (mutation of L14Y in TatAd) and *tatAyCy* gene in pTAT2.0, Km^R	This study
p90S-2.0	Harboring *tatAdCd*-90S (mutation of P90S in TatCd) and *tatAyCy* gene in pTAT2.0, Km^R	This study
p13Y-2.0	Harboring *tatAdCd* and *tatAyCy*-13Y (mutation of V13Y in TatAy) gene in pTAT2.0, Km^R	This study
p93S-2.0	Harboring *tatAdCd* and *tatAyCy*-93S (mutation of P93S in TatCy) gene in pTAT2.0, Km^R	This study
p14Y-2.0AS1	Harboring *npas* gene in p14Y-2.0, Km^R	This study
p90S-2.0AS1	Harboring *npas* gene in p90S-2.0, Km^R	This study
p13Y-2.0AS1	Harboring *npas* gene in p13Y-2.0, Km^R	This study
p93S-2.0AS1	Harboring *npas* gene in p93S-2.0, Km^R	This study

Table S2 Nucleotide sequence of primers

Oligonucleotides	Nucleotide sequence (5′ to 3′)*
Amys113-F	TATGGATCCATGCTGACGC
Amys113-R	TTAGGCGATTTCCAGCCAC
GluR-F	ATGGATAGGAGGGATAATGGAGGC
GlmU-45R	CATGGATCCATGTCCAACTATTGTGACAA
GlmU-104R	GCCGGATCCGGCTTCCATCGTTTCGGCAG
GlmU-124R	GAAGGATCCTTCCGCTACAGCGGTTAAAA
318-R	TCGGTTCCCTCCTCACTTTTC
318-F	TTACAGGATCCTCTAGAATTCCCG
L14Y-F	CTCATCTTGGTT**TAT**GTAATTGCGCTG
L14Y-R	CAGCGCAATTAC**ATA**AACCAAGATGAG
V13Y-F	TTTATTTTAATT**TAT**GTGGTGGCGCTT
V13Y-R	AAGCGCCACCAC**ATA**AATTAAAATAAA
P93S-F	GCTTTCATCAGC**AGC**GGGCTGTATGAA
P93S-R	TTCATACAGCCC**GCT**GCTGATGAAAGC
P90S-F	CGGTTTGTGGCG**AGC**GCGCTGACCGAC
P90S-R	GTCGGTCAGCGC**GCT**CGCCACAAACCG
Acd-F	TGTCTGCAGACATCTTTGGCGTGGAGT
Acd-R	ATGAGATCTCATTATTTCTGATTTTTTCATTTCAGGC
Acy-F	GCCGGATCCGGCATTAAAGGCATTCTTAATTTCAC
Acy-R	TGCCTGCAGGCAGCCTATAATATCAGCATCTCC
Acy-f1	CGCTCATAATCTACCTTCAGCAGACGGATG
Acy-r1	CATCCGTCTGCTGAAGGTAGATTATGAGCG
Acy-f2	CTTTCATCAGCCCTGGGCTGTATGAAAAAG
Acy-r2	CTTTTTCATACAGCCCAGGGCTGATGAAAG
SecDF-Up1	ATATCTAGATATGAAGTCCAGCCTGCCA
SecDF-Up2	CACGGCAGCAATGAAAGTATCAAGCCAGATGACGAGC
SecDF-Dn1	GCTCGTCATCTGGCTTGATACTTTCATTGCTGCCGTG
SecDF-Dn2	GAAGGATCCTTCCAGACAAGCCATAGTTG
SecDF-UpR	CGCCATGTCTTTTGCTTCCT
SecDF-DnF	TGAATACAAGATGGCGATTG
SecDF-F	AATGTGAAAGGAGCGTGG
SecDF-R	CGACAGTCAGCACCGTATTA
T2-MCS2F	GCAAGCAGCAGATTACGC
T2-MCS2R	ATGTGATAACTCGGCGTA
ACd-up1	TCGTTGATTTTGACAATCCG
ACd-up2	GAGACTGAAGACACAGTCCGTCTTTGCTGTCATCCTTTCATTGATT
ACd-dn1	AATCAATGAAAGGATGACAGCAAAGACGGACTGTGTCTTCAGTCTC
ACd-dn2	GCTGGATCCCGACCATTTGCTCATATTCC
ACd-dnF	CTCAGATGTGCTGGTGATCG
ACd-upR	AATGATCAGCGCAATTACCA
ACd-F	CAACATCGTCGTCCTTACCG
ACd-R	ATTCCAAAGCGGTGCTTAAG
ACy-up1	AAACGACGAACGTAACGCTT
ACy-up2	CCATTCTGAAAAAAGAATGGCTCAAAGCCTGCCCCCTTTCCTT
ACy-dn1	AAGGAAAGGGGGCAGGCTTTGAGCCATTCTTTTTTCAGAATGG

ACy-dn2	ATGGGATCCTACGGCAATGGGATTCACAT
ACy-dnF	CGTGCCATTGCTCATTCTTTA
ACy-upR	GAATATCAAAAGCGCCACCA
ACy-F	ATGCCGTCAAAGTCGATTCC
ACy-R	TCTGTTCAGTTCGGTGCTCT

*Underlined represented restriction site. The bold sequence encoded a mutated amino acid.
